# Rapid, parallel path planning by propagating wavefronts of spiking neural activity

**DOI:** 10.3389/fncom.2013.00098

**Published:** 2013-07-18

**Authors:** Filip Ponulak, John J. Hopfield

**Affiliations:** ^1^Brain CorporationSan Diego, CA, USA; ^2^Department of Molecular Biology, Princeton UniversityPrinceton, NJ, USA; ^3^Institute for Advanced Study, Princeton UniversityPrinceton, NJ, USA

**Keywords:** path planning, navigation, parallel processing, mental exploration, wave propagation, spike timing dependent plasticity, hippocampus, neuromorphic systems

## Abstract

Efficient path planning and navigation is critical for animals, robotics, logistics and transportation. We study a model in which spatial navigation problems can rapidly be solved in the brain by parallel mental exploration of alternative routes using propagating waves of neural activity. A wave of spiking activity propagates through a hippocampus-like network, altering the synaptic connectivity. The resulting vector field of synaptic change then guides a simulated animal to the appropriate selected target locations. We demonstrate that the navigation problem can be solved using realistic, local synaptic plasticity rules during a single passage of a wavefront. Our model can find optimal solutions for competing possible targets or learn and navigate in multiple environments. The model provides a hypothesis on the possible computational mechanisms for optimal path planning in the brain, at the same time it is useful for neuromorphic implementations, where the parallelism of information processing proposed here can fully be harnessed in hardware.

## Author summary

Humans and animals can quickly and reliably solve spatial navigation and path planning tasks. However, neural mechanisms underlying these processes are not completely understood. Discovery of, so called, place cells—the hippocampal cells getting activated whenever an animal enters a certain spatial location—gave rise to the idea that the hippocampus contributes to the creation of internal, neural representations of the environment. Here we demonstrate that spatial navigation can rapidly be solved in the hippocampus-like neural network by parallel mental exploration of alternative routes. A possible biological mechanism to implement parallel exploration is through propagating waves of neural activity spreading across the entire network representing a given environment. We present a model, where such waves of spiking activity alter synaptic connectivity through spike-timing-dependent plasticity and create a vector field, which can guide an animal through the environment to selected target locations. In a set of computational experiments we demonstrate that planning can be solved during a single wavefront passage through the network. Moreover, the model is capable of suggesting an optimal solution for multiple competing targets, and it can embed multiple environments for trajectory planning.

## Introduction

One of the central problems for neurobiology is to understand the computational effectiveness of the brains of higher animals. Brains rapidly carry out extraordinary feats of visual scene analysis or problem solving through thinking on “wetware” that is tens of millions times slower than modern digital hardware. Part of the explanation is brute-force *anatomical* parallelism.

In this paper we develop a model of parallel computational processing in the context of path planning and spatial navigation. We propose that spatial navigation can be solved through simultaneous mental exploration of multiple possible routes. A typical mental exploration task for an animal might involve knowing an extensive terrain containing a few water sources, being motivated (being thirsty) to seek the nearest water source. Hopfield (2010) recently described a way that serial mental search for a useful route could be done by a moving clump of activity and synapse modification in a hippocampus-like neural network^1^. We show here that a best path can rapidly be found by *parallel* search in the same kind of network, but by a propagating wave of spiking activity. The process of path planning and navigation, as proposed in our model, consists of the following steps: (1) expanding waves of neural activity are initiated from the place cells corresponding to selected target location(s); (2) the propagating waves alter synaptic connectivity within the network through spike-timing-dependent plasticity and create a directed synaptic vector field (SVF) converging on the goal locations; (3) this vector field is used by an animal to navigate toward targets; (4) whenever a new planning process is necessary, all synapses are reset to the baseline state and waves of activity can be initiated from the new target locations.

Can animals employ such parallel mental exploration to solve novel problems? Indeed can humans do so? Recent electrophysiology experiments demonstrated existence of expanding, traveling waves of neural activity in the hippocampus, associated with theta-oscillations (Lubenov and Siapas, 2009; Patel et al., 2012), as well as with much faster sharp wave ripples (Ellender et al., 2010), yet, no link between such waves and spatial planning has been shown so far.

One of the major roles of theory is to elucidate interesting consequences and possibilities inherent in our incomplete experimental knowledge of a system. The fact that hippocampus-like neural substrate can support parallel mental exploration, as explored here, is such a possibility. New experimental paradigms could easily test for parallel mental exploration in rats. These ideas also form the basis for novel neuromorphic circuits in engineering, which could be used to implement effectively certain Artificial Intelligence algorithms such as those based on the idea of a wave-front propagation (Dorst and Trovato, 1988; Dorst et al., 1991; LaValle, 2006) by taking advantage of the true parallelism of the neuromorphic hardware systems (Boahen, 2005; Misra and Saha, 2010).

## Results

We consider like (Hopfield, 2010) a network of excitatory “place” cells for a very simple model animal. Through experience in an environment, each cell has learned synaptic connections from a sensory system (not specified here) that make it respond strongly only when the model animal is near a particular spatial location. These response place fields are our modeling equivalent of the response place fields observed in the rodent hippocampus (O'Keefe and Dostrovsky, 1971). For display purposes, the activity of each place cell can be plotted at the spatial location of the center of the receptive field corresponding to that place cell. In such a display there is a localized activity clump surrounding the actual spatial position of the model animal. When the animal moves, this activity region follows the location of the animal. If an animal wanders throughout an environment over an extended time, the synaptic plasticity will result in excitatory synaptic connections being made only between cells that are almost simultaneously active (Hebb, 1949). If the exploration process is not systematically directional and is extensive, connections will on average not have directionality. The CA3 region of the hippocampus has such intra-area excitatory connections with the requisite spike-timing-dependent plasticity, or STDP (Amaral and Lavenex, 2006).

The fundamental neural network to be studied is thus a sheet of place cells, each having excitatory connections to the others with centers within its receptive field footprint, but not to distant neurons. Experimental support for the existence of such connections (direct or indirect) comes from the coordinated phase-change-like response of place cells, trained in two environments, experiencing a visual environment that mixes the two environments (Wills et al., 2005).

The model neurons considered in our study are of the integrate-and-fire type with a short dead-time and spike-frequency adaptation (implementation details are provided in the Methods section at the end of the paper).

**Figure 1:**
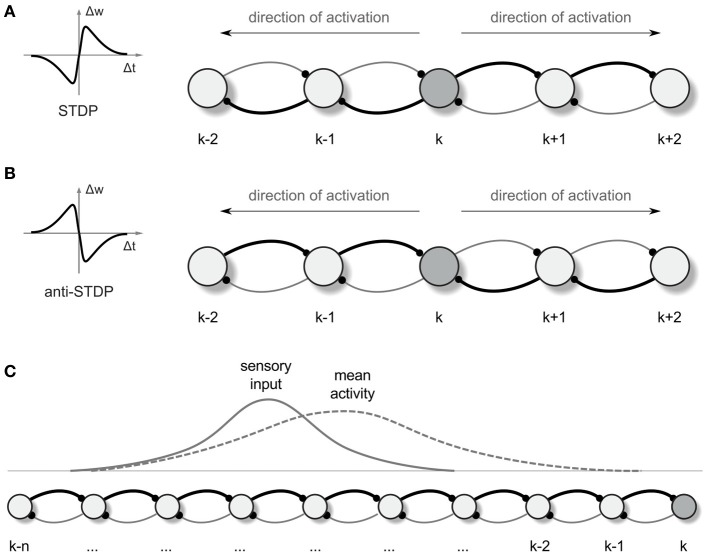
**Synaptic vector field formation. (A,B)** Illustration of the synaptic strength changes in a one-dimensional network altered by “causal” STDP **(A)** and “anti-causal” STDP **(B)** after a neural activity was propagated from the neuron k in the directions denoted by the arrows. The connections are shown as arcs with the direction of connection denoted by little dots representing synapses. Stronger connections are represented by the thicker lines. Left panels are the schematic illustrations of the synaptic weight changes Δ*w* as a function of the time lag Δ*t* between the post and presynaptic spikes, for STDP **(A)** and anti-STDP **(B)**. **(C)** Due to the asymmetry in the strength of connection from- and to- any particular neuron in the network, the mean neural activity observed in the network is shifted with respect to the input current distribution.

We investigate whether and how the described setup can implement parallel search for optimal pathways in the environment represented by the neural network. Because we rely on simulations of a system whose mathematics we cannot fully analyze, it is sensible to present a line of argument that develops insight about expected behaviors. Consider a simplified model comprising of a line of neurons, each reciprocally connected to its two nearest neighbors (cf. Figures 1A,B). With specific parameter settings, a single spike can initiate an activity pattern that consists of a pair of spikes marching from the initiation site toward the ends of the line at constant speed, one in each direction (Aertsen et al., 1996). In a system with intrinsic neuronal adaptation, there is a dead time before another pair can be propagated in this same region.

**Figure 2:**
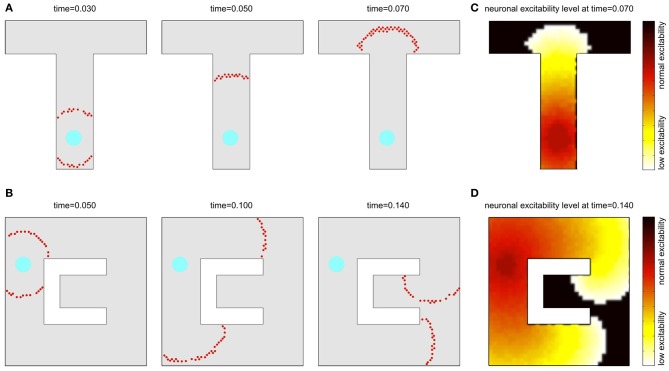
**Wavefront propagation and neuronal adaptation.** Illustration of a wavefront propagation in a network of synaptically connected place cells for two different environments **(A,B)**. Cyan fields are the initiation points of the wavefronts. Red dots are the action potentials that occurred in a time window of 0.002 s centered at the times indicated. Plots **(C,D)** show color maps of the average level of a neural adaptation in the particular regions of the network after a single wavefront passage up to the states illustrated in the right-far plots in **(A,B)**, respectively. Brighter colors in these maps represent lower excitability of the neurons at the corresponding locations.

A similar phenomenon can be observed also in a two-dimensional sheet of neurons with recurrent local connections over a small but extended region. In an example presented in Figures 2A,B, the synaptic connection strengths are chosen so that a few pre-synaptic cells must spike almost simultaneously to fire the post-synaptic cell. Seeded with a few approximately synchronized firings of nearby cluster of neurons, a propagating circular wavefront of activity is observed in which each neuron fires only once (Kumar et al., 2008). A second wavefront cannot be initiated in a region that the initial wavefront has traversed until the adaptation has decayed (cf. Figures 2C,D). Note, that although in our model we consider a single-spike activity, the basic activity events propagated through the network may in principle also consist of short bursts of spikes, which is biologically more realistic in the context of the hippocampal cell activity.

Propagating wavefronts can have profound effects on synaptic modifications through STDP. Consider again a one-dimensional network as illustrated in Figure 1. Any non-symmetric STDP rule will produce, in one dimension, synaptic change patterns that display whether the “front” of activity that went by was going toward the left or toward the right. Normal or “forward” STDP which enhances synapses at which the pre-synaptic spike comes before the post-synaptic spike will result in rightward-going synapses being stronger than leftward-going synapses if the wavefront passes moving to the right (Figure 1A). “Reverse” or “anti-” STDP which enhances synapses at which the pre-synaptic spike comes after the post-synaptic spike (Bell et al., 1997; Kampa et al., 2007; Roberts and Leen, 2010) will result in leftward-going synapses being stronger than rightward-going synapses if the wavefront passes moving to the right (Figure 1B). The same basic idea intuitively extends to two dimensions, where STDP results in synaptic change that can be interpreted as a vector field (in the following we shall call it a synapse vector field or SVF), showing the orientation of the propagating wavefront that caused the synaptic change. In all our simulations we use reverse STDP induced by propagating spike wavefronts that creates an SVF pointing toward the center (initial point) of the waves. Our use of reverse STDP is motivated by certain conceptual and technical advantages of this approach over regular STDP, as it will be described later in the paper.

### Simple path planning problem

Consider for definiteness the “T” shape environment shown in Figure 2A. We presume that by exploring the environment, each neuron has acquired a place field such that it is driven strongly only when the simulated animal is near the place field center and the drive to the cell falls off smoothly away from that location. For display purposes, in all figures the cells are arranged so that whatever property of the cell is being plotted, its (*x,y*) plot location is the location of its place field center. The receptive fields considered in our experiments are assumed to have Gaussian shapes and to cover 25–50 cells in their footprints in a simulation using a network with 2000 place cells. In such a setup, if an animal explores an environment, synapses with simple STDP will form strong connections between neurons with similar place fields, i.e., between neurons that are close together. To this point, the general approach is like that previously used in Hopfield (2010).

Imagine that the simulated animal, in exploring an environment, finds a target T, such as a source of water, to which it may later want to return. Let the dendrites of the place cells in the vicinity of T become connected to axons from an “exciter” which, when activated, can briefly drive these place cells to fire. Such activation will result in an outgoing wave of single spike activity emanating from T as center as illustrated in Figure 2A (where the cyan field represents the T location). This wave will spread until every neuron has fired an action potential. As noted before, the next wavefront is possible only after the neural adaptation fades away. Also, to prevent runaway, we use a global inhibitory mechanism, where inhibition is proportional to the network activity, resulting in a balanced excitation-inhibition (for more details we refer to the Methods section).

**Figure 3:**
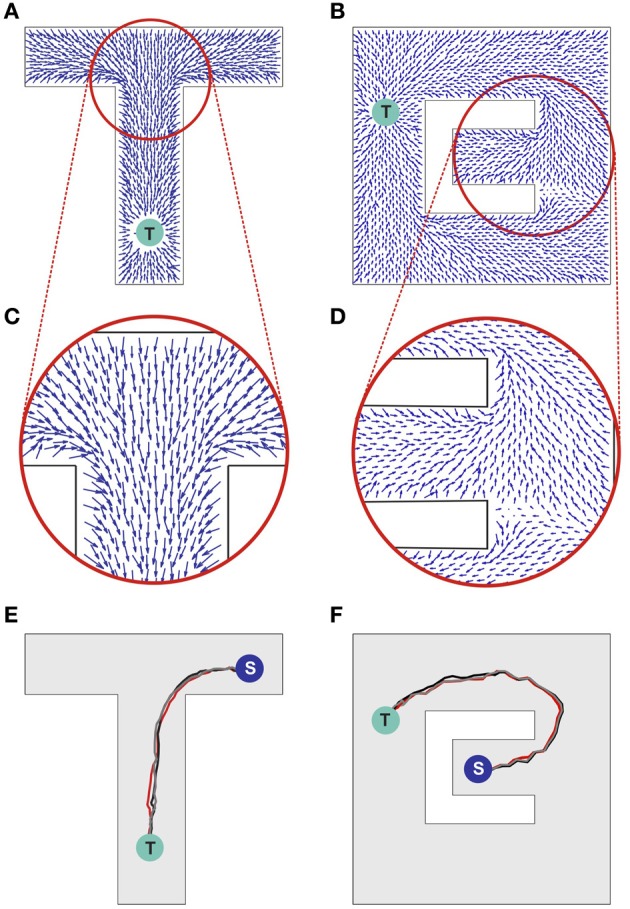
**Synaptic vector field and spatial navigation. (A,B)** Synaptic vector fields resulting from the wavefront initiated at point T and propagated as illustrated in Figures 2A,B, respectively. **(C,D)** The insets show details of the vector fields around the bifurcations in the simulated mazes. **(E,F)** Typical movement trajectories observed in the considered models resulting from the vector fields from **(A,B)**, respectively. The trajectories begin in points “S” and end in the target locations “T.” For additional results see also Movie S1 in supplementary materials.

The propagating wave and the asymmetric synaptic plasticity implicitly define a vector field, which represents the local direction of the wavefront, i.e., the vector is normal to the wavefront and points in the direction of propagation. We should define what is meant by “shortest path” or more generally “optimal path” for present purposes. While the synapse vector field is defined only at the discrete locations of place cell centers, the synapses themselves will be used to control the continuous motion of an animal in real space. The discreteness of the place cell representation will contribute fine-scale noise in the actual physical path. The optimality we are interested in is macroscopic optimality—for example, choosing the right way to go around an obstacle. The physical pathlength contribution introduced by jitter from the discreteness of the neural representation is not of interest. Before the single-spike activity wave was initiated, all directions were equivalent, and the SVF was zero everywhere. Afterward there is a local directionality, because the timing of pre-post spike pairs depends on the spatial separation of the pairs projected on the direction of wavefront propagation. Sample SVFs that result from the anti-STDP rule are shown in Figures 3A–D. Here the vector fields are illustrated using directed arrows originating from the preferred locations of each place cell in the network. The direction and the length of each arrow represent, respectively, the direction and the strength of the vector field in a given location (see Methods for details).

The SVF can be used for finding the shortest pathway to the location being the source of the propagated wave. Intuitively, since the first wave to arrive at your position comes via the fastest path, if you simply backtrack, always going backward along this vector field, you will reach the target by the shortest path. In either case, the synapse vector field contains the information necessary to find the shortest route to the target. It is merely a question of following the vector field forward (anti-STDP) or backward (STDP).

### Obtaining motor commands for following the synaptic vector field

For illustrating an idea of how the SVF can be used for guiding an agent (a simulated animal or a robot) movement, we return to the one-dimensional case (Figure 1C). In 1 dimension, if the propagating wavefront has passed by locations *k* through *(k-n)* while moving leftward, and the anti-STDP rule has been applied, rightward-directed synapses [e.g., *(k-1) → k*] are strengthened more than leftward ones (*k* → (*k*-1)). Before this process, if the animal was located at a particular location in space, a bump of place cells would be active, symmetrically located around that location. In the presence of the asymmetric synapse modification, the bump of activity is biased and no longer centered on the actual physical location (Figure 1C; cf. Levy, 1989; Blum and Abbott, 1996). This bias can be converted into a motor command proportional to the bias and pointing toward the direction of a wavefront passage.

Precisely the same problem occurs in earlier work on a hippocampus-like model of actions based on “thinking” (Hopfield, 2010). In that model there were two clumps of activity, one representing the present position of the animal and the other representing where the animal thought it should be a short time later. The difference between the locations of these two clumps was used to produce motor commands that moved the animal toward the desired future location. The model was fully implemented with spiking neurons.

Since the task of generation of motor command is not the major focus of our paper, here we use a simplified approach. Namely, we assume that a receptive field corresponding to the present animal location is activated by applying tonic excitation to the corresponding place cells and then any place cell firing a spike causes a pulse of force moving an agent toward the preferred location of that cell. The asymmetry in the weight configuration around the receptive field results in a higher probability of firing of those adjacent place cells that are located along a direction of a vector field. As a consequence an agent moves to a spatial new location along the optimal pathway. The details of the algorithm are provided in the Methods section at the end of the paper.

Sample movement trajectories resulting from applying the described procedure to a simulated animal are shown in Figures 3E,F (see also Movies S1 and S2 in Supplementary materials). These trajectories result from the SVFs illustrated in Figures 3A,C and Figures 3B,D, respectively. In particular, Figure 3F illustrates the shortest path aspect of the available information—because the target is located above the midline, the wavefront arrives at the branch containing the animal at S from above before the wavefront from below (cf. Figures 2B, 3B). Neural adaptation prevents the wavefront arriving from below from penetrating this region. Thus, the SVF leads to a route from S to T going upward.

Notwithstanding the fact that the algorithm used here is not providing details on the possible neural implementation of action execution, it is important to emphasize that the actions are triggered by individual spikes and hence each spike contributes to the agent behavior change. The average population activity pattern determines the mean movement trajectory along the vector field, whereas the particular spikes add some stochasticity to the behavior (reflected, e.g., in a small trial-to-trial variability of the movement pathways observed in Figures 3E,F). Such stochasticity has some advantage in certain situations. For example, it may be useful for avoiding local minima, or for selecting one choice when several alternatives have equal probability.

### Navigation in an environment with multiple targets and values

**Figure 4:**
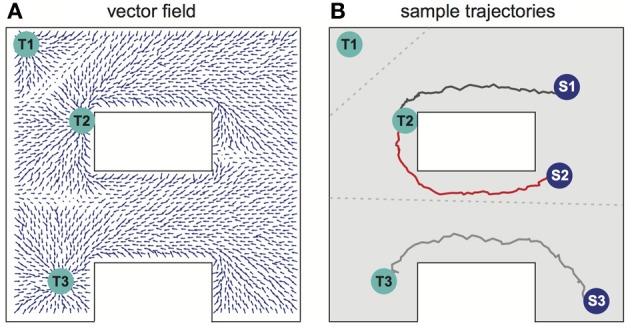
**Navigation in a system with multiple targets. (A)** Synaptic vector field created in the network with targets in locations Tl, T2, T3. **(B)** Typical movement trajectories observed in the system for the initial agent locations as indicated by spots Sl, S2, S3. The path selection and the path shapes are determined by the shape of the vector field and by the initial agent location's. The vector field has three basins of attraction corresponding to the particular targets—the bounds of the basins of attraction are indicated by the gray dotted lines. For additional results see also Movie S2 in supplementary materials.

Several different relevant targets might be simultaneously available in an environment. For simplicity, the case when all targets have the same intrinsic value is first considered. Figure 4A shows the SVF that results when single spike propagating circular waves simultaneously originated at three targets. Because the single-spike wavefront cannot propagate into a region that another wavefront has recently traversed, any subregion is therefore traversed by only a single wavefront, the one that arrives first, and is thus closest to its source. Within that subregion, the vector field is the same as it would have been if only the source responsible for the traversing wave had been present. The three subregions of the three possible targets of Figure 4A are shown in Figure 4B (compare to Movie S2 in the Supplementary materials). Which target is nearest, and thus should be navigated to, depends on the current location of the agent. The same figure illustrates the paths followed for three possible initial agent locations. Note that the SVF is defined everywhere, independent of the location of the agent when the wavefront is generated.

When multiple targets are present, an optimal choice will involve balancing the cost due to the length of a path and the reward that will result if that path is followed. For a single target, the net reward due to following a path of length *L* is *R–CL*, where *C* is the cost per unit length of following any path, and optimizing net reward simply minimizes *L* over the set of possible paths. When multiple targets of equal value are present, the same net reward expression applies, but the set of relevant paths over which a minimum is sought includes paths to each possible target. Accordingly, if the targets all have equal value, the described procedure selects the target that can be reached by the shortest possible route.

Now suppose that different possible targets *T*_*k*_, *k* = 1, have different rewards *R*_*k*_. When all wavefronts propagate with the same velocity, it is useful to think in terms of times rather than lengths. *R*_*k*_ then can be seen as an effective shortening of the time to navigate to a reward. A simple way to implement it is to initiate wavefronts first at the target locations corresponding to greater rewards and later at the locations with lower rewards. The introduction of these differential delays represents the value differentials between the various targets. These delays shift the boundaries of the regions such as those of Figure 4B in a way that represents the differing values of the target. The optimal relative initiation times can be learned on the basis of maximizing the term (*R*_*k*_–CL). For any winning target, the path followed to that target is the same as would have been used if that target alone were present.

### Dealing with noise

**Figure 5:**
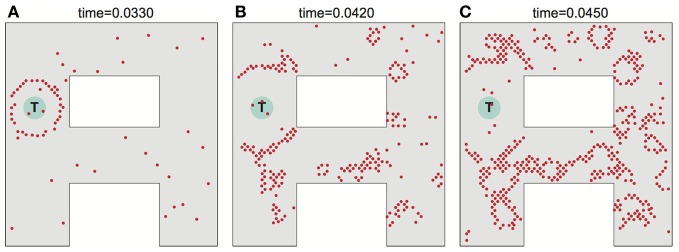
**Effects of noise on wavefront propagation. (A)** A single wavefront is initially started from the point T. Noise results in spurious single spikes or missing spikes. When spurious spikes cluster, they can serve as initiation sites for new circular waves centered at locations where there is no target. In addition, spurious and absent spikes cause irregular wavefront propagation or even wavefront extinction as illustrated in **(B,C)**. Network activity shown at times as indicated. The noise is modeled by injecting spike currents to randomly selected neurons at random time steps.

Noise can adversely affect the ability of the network to propagate a wavefront in the ideal fashion to set up the desired synaptic field. Figure 5 illustrates what can happen when noise is severe. Spurious single spikes are generated, and spikes can fail to occur. When spurious spikes cluster, they can serve as initiation sites for new circular waves centered at locations where there is no target. In addition, spurious and absent spikes cause irregular wavefront propagation or even wavefront extinction.

The major noise issues concern setting up the SVF. Once it is set up, the motor control system effectively averages over the vector field in a small region, and noise in following the SVF is not a major issue.

Having a large system is the first defense against noise. As the system size grows, the number of neurons making synapses onto a particular cell, which must be simultaneously active to initiate a spike in that cell, can be increased, and the likelihood of spurious single spikes decreases. The likelihood of a spatial cluster of spurious spikes being large enough to trigger a new wavefront is also reduced. There is considerable latitude for exploiting the large number of cells available in real neurobiology.

There are also cellular means to suppress the effect of noise. Set the threshold for spike generation at some particular level, and consider the ability of *N* cells connected to this one to trigger it to spike when a passing wavefront goes by. There will never be exact synchrony in the firing of the presynaptic cells, so while *N* cells firing may typically be required to fire the cell, less than *N* may also sometimes do so, and more than *N* may fail to fire it. Reliable wavefront propagation is enhanced by any biophysical effect that sharpens this threshold on *N*. One way to sharpen this threshold is by determining whether a particular neuron in a network is excited by spikes coming from a small number of neurons being unusually effective (for example because of noise), or by a larger number of neurons with typical effectiveness. A method of making this distinction can be implemented in a biologically realistic way by using supra-linear spatial summation, a phenomenon observed in biological neural circuits (Nettleton and Spain, 2000; Urakubo et al., 2004). In our work we use a simple phenomenological model of such a supra-linear integration that favors weak excitation from multiple inputs over strong excitation from a few inputs. This is achieved through a non-linear summation of synaptic input currents to the neuron, such that the effectiveness of presynaptic spikes is increasing with a number of simultaneously active inputs to the neuron (see Methods section for details). Although, in this algorithm the appropriate setting of the neuron activation threshold is still important, it is no longer a critical factor for the problem at hand. With this approach more emphasis is put on how many presynaptic neurons are active simultaneously, rather than how strong the particular connections are. In this way the algorithm works better than the threshold algorithm for networks with greater heterogeneity of synaptic connection strengths.

### Navigation in multiple environments

When a rat is familiar with multiple environments, a particular hippocampal neuron can have place fields in more than one environment, with no apparent coordination between them (Bostock et al., 1991; Wilson and McNaughton, 1993). We also therefore investigate whether our network model can learn and effectively perform navigation in multiple environments when each neuron has a place field in each environment. When the place cells in one-environment and place cells for a second environment are uncorrelated, the synaptic connections needed in both environments can be simultaneously present. If the number of neurons is sufficiently large, when the sensory signals come from one environment there is little crosstalk between the representations of both environments, and the presence of the second set of synapses simply inserts a modest level of noise. One can similarly anticipate that single spike wavefronts can be initiated and will propagate in any particular environment when multiple environments are known. The wavefronts will produce a vector field that can later be used to guide the animal in this particular environment. This is a significant extension, for without it each neuron needs to be specific to a single environment, which would be both inefficient and not in correspondence with biology.

**Figure 6:**
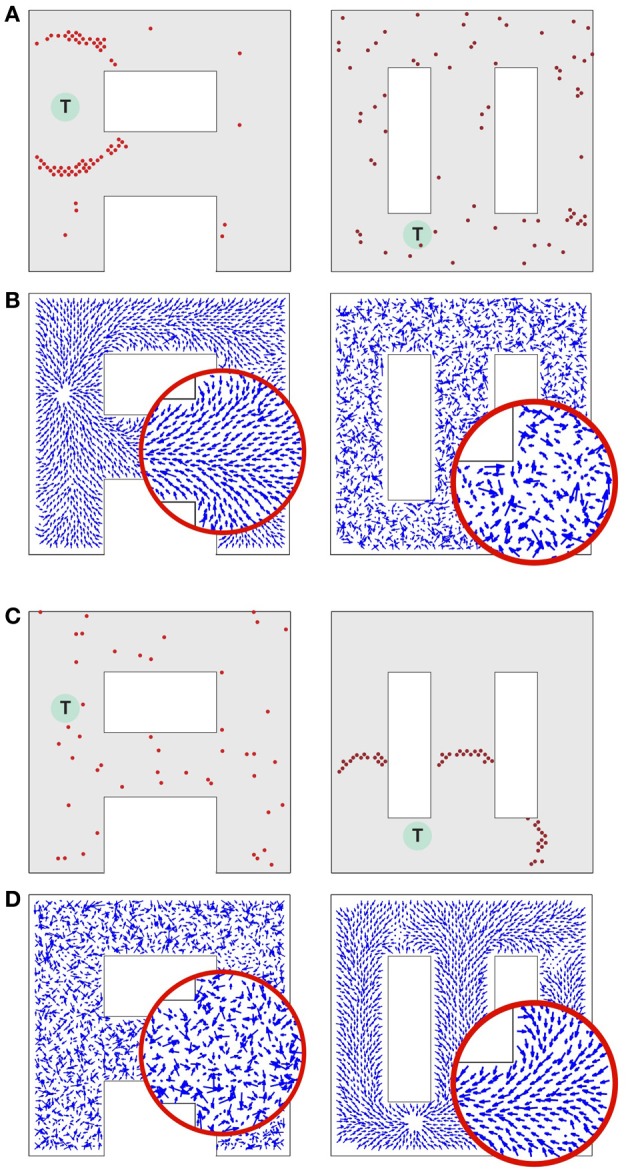
**Synaptic vector field formation in multiple environments.** (**A**, left) Wavefront propagation in environment “*A*” short time after the activity wave initiation at the target T. (**A**, right) The same activity pattern as in (**A**, left), but displayed in the “∞” environment plotting representation. (**B**, left) Synaptic vector field resulting from the propagation of a wavefront illustrated in (**A**, left). Note a single attractor corresponding to the location T, that is the center of the wavefront. (**B**, right) The synapse vector field due to the same synapse changes as in (**B**, left), but calculated using the positions of the neurons in the “∞” environment. **(C,D)** The same plots as for **(A,B)** except that the wavefront has been initiated at target T in the “∞” environment. All results are qualitatively like those in **(A,B)**, except that the roles of the two environments are reversed. Synaptic vector fields in plots **(B,D)** are visualized using the same normalization factor (arrow scale) for both environments.

Consider a network that is supposed to operate on two different environments as illustrated in Figure 6. Due to their shapes we call these environments “A” and “∞.” While in the rat many place cells would be specific to one environment, such specificity reduces the crosstalk between the environments, and de-emphasizes the crosstalk effect we wish to evaluate. Here, however, we assume that each place cell represents the animal's locations in both environments.

A spike generated in any cell will produce excitatory postsynaptic potentials in all its neighbor cells in one environment and all its neighbor cells in another environment. As in the previous experiment, the model parameters are set such a single spike cannot cause action potentials in the postsynaptic neurons. As before, supra-linear summation helps to promote stable propagation of the existing wavefronts, and to prevent single, isolated spikes from producing new wavefronts.

Consider a network activity caused by the simultaneous excitation of a certain set of the topologically nearby cells in the environment “A.” When a plot is made with each cell located at the preferred location it represents in environment “A,” the dynamics of this neural activity will be seen as a wave propagated through the network (Figure 6A, left). The same activity observed from the perspective of the “∞” environment (that is by reorganizing the network by putting place cells at the locations they represent in the “∞” environment) would appear as a random network activity (Figure 6A, right). Since the spikes observed in the “∞” environment appear sparse, they are unlikely to initiate a wavefront in this representation. Similarly, at any particular moment while a wavefront in the “A” environment is propagating, the synaptic connections representing the “∞” environment introduce drive to neurons that should not be driven at that moment. Occasionally such neurons can produce crosstalk-induced spurious spikes (cf. solitary spikes in the left panel in Figure 6A, occurring far away from the wavefront).

**Figure 7:**
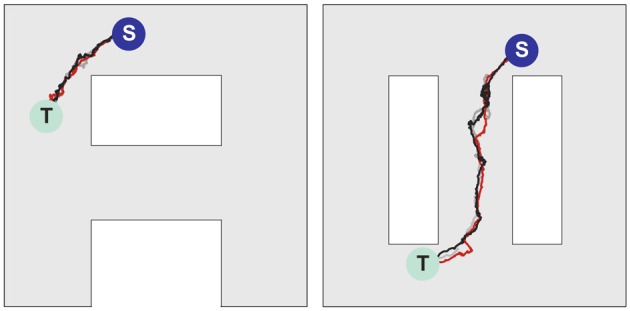
**Navigation in multiple environments.** Sample movement trajectories in the environment *“A”* **(left panel)** and “∞” **(right panel)** resulting from the synaptic vector fields shown in **(left)** and **(right)**, respectively. Three different trials for each environment are illustrated. The trajectories start in the S locations and end in the T locations.

Figures 6B–D illustrates that at the level of two environments and around 2000 place cells, there is little effect of crosstalk on the ability to function in each environment as though the other did not exist. Figure 6B (left) shows that the SVF induced by a wavefront initiated at T (cf. Figure 6A, left) develop as expected, representing a flow back toward the target from all points in the “A” environment. Figure 6B (right) shows the SVF for the same synaptic changes, but calculated for the place cell locations in the “∞” environment. Here the vectors point in random directions because there is no spatial organization to the synapse change in this representation. The same kind of result is obtained when the wavefront is initiated in the “∞” environment as in Figures 6C,D with the roles of the two environments reversing. In each case the vector field created by the single-spike wavefront successfully navigates an animal from a starting point in the given environment to the target as illustrated in Figure 7.

## Discussion

The problem of planning and executing a complex motion over a protracted time-period which will optimally take an autonomous agent from its present location and configuration to a desired target location and configuration is common to both animal behavior and robotics. In its simplest manifestation there is only a single target, a single known environment, and a short or fast path is preferred over a longer or slower one. The trajectory planning must accommodate the physical constraints posed by the environment. Additional complexities might include the simultaneous presence of multiple targets, possibly of different intrinsic values, terrain which affects the value of trajectories in a non-trivial fashion, and multiple environments.

The neurally-inspired network presented in our work has been shown to solve the planning problem in several steps. First, in an exploratory phase it learns an environment by developing a set of “place” cells whose locations reflect all possible trajectory boundaries due to kinematic constraints or constraints in the behavior arena. It develops in this exploration process interconnections between all pairs of places that can be visited in temporal contiguity, and thus can be possible candidates for a section of a trajectory. Second, given the expected set of synaptic connections, the excitation of a target location (or locations) initiates a wavefront of single spike- or single burst activity that propagates outward from the initiation site(s). The wave propagation process is terminated when a wavefront reaches the present location of the agent. The passage of such a wavefront produces synapse modification pattern that can be described as a vector field. The desired trajectory is simply the path along the SVF from the present location to the (or a) target. Since the SVF lines are produced by an expanding circular wavefront, they converge when followed backward toward a source, and thus provide stable guidance for going to a target location.

The full extent of the parallelism available in our concept is perhaps best illustrated in Figure 4. The system simultaneously selects the closest target and the best route to that target from a single propagation of the exploration wave. Conventionally, a best path would be found for each target sequentially, using a serial algorithm to rate possible paths, and a choice of target then made between these optimal single-target paths. The conceptualization of the parallel search method and the demonstration by simulation that best trajectories can be followed in neuromorphic simulation are the major accomplishments of this paper.

### Network analysis

As mentioned before, the goal of our paper was to present a concept of parallel exploration through propagating waves of neural activity and STDP-altered SVFs. We have illustrated our concept in a set of simulations, but we have not attempted to quantify our results. An interesting extension of our work thus would be to perform an analysis of the properties of our system. Interestingly, such an analysis has recently been offered for the network proposed in (Hopfield, 2010), which is of the same type and topology as the one considered in our work. Indeed, Monasson and Rosay (2013) provided an indepth theoretical analysis of the dynamics and storage capacity of that network as a function of such parameters as: network size, level of neural activity, level of noise, or size of place cells. Specifically, using the statistical mechanics tools, the authors analysed conditions necessary for the network to learn multiple maps (environments). The storage of a map manifests itself through the fact that the neural activity is localized, and acquires a clump-like shape in the corresponding environment. Remarkably, according to the analysis performed by the authors, a moderate level of noise can slightly increase the capacity storage with respect to the noiseless case. However, when the number of environments or the noise are too high the neural activity cannot be localized any longer in any one of the environments. For high noise, the activity, averaged over time, becomes uniform over space. For high loads the activity is not uniform, but is delocalized with spatial heterogeneities controlled by the cross-talks between the maps. The paper provides quantitative results for the transition between these states. The authors also analyse storage capacity of the network, that is a maximum number of environments for which a stable representation of a given environment can still be retrieved, as a function of network size and topology. For the network of the type considered in (Hopfield, 2010), and so also in our work, the storage capacity is proportional to the network size and is estimated to be of the order of 10^−3^ bits per synapse (for the 2 dimensional space representation and under the optimal conditions). Interestingly, these results are consistent with an earlier analysis for a network with a similar topology but with a different neuron type given in Battaglia and Treves (1998).

### Related models

The wave-propagation concept has first been introduced by Dorst and Trovato as an efficient parallel method for path planning (Dorst and Trovato, 1988; Dorst et al., 1991) and since then has widely been used in robotics and computer science (LaValle, 2006). The wave-front methods are essentially the same as exhaustive or heuristic versions of a classical A^*^ search algorithm (Dijkstra, 1959; Hart et al., 1968) of whose optimality is proven. Several neural models for spatial navigation using the concept of propagating waves have been proposed so far (for reviews see, e.g., Lebedev et al., 2005; Qu et al., 2009). However, only a few models addressed a question on how the propagating neural activity can be transformed into an appropriate configuration of synaptic connectivity able to later guide an agent to a target location (Roth et al., 1997; Gorchetchnikov and Hasselmo, 2005; Qu et al., 2009; Ivey et al., 2011). To the best of our knowledge, our model is the first one to demonstrate that biologically plausible, temporally asymmetric synaptic plasticity rules can achieve this goal. Also, most of the previous models assumed multiple trials for learning a complete set of optimal paths for every new selected target location. In contrast, in our model, once an agent becomes familiar with an environment, a single passage of an activity wavefront through the network is sufficient to create a SVF guiding an animal from any possible location in the experienced environment to a target location. Interesting enough, such an ability of animals to rapidly replan routes if the starting and goal points are changed to new, random locations within a known environment has recently also been observed experimentally (Pfeiffer and Foster, 2013).

### Biological relevance

Parallel exploration as proposed in our model requires mechanisms that support stable propagation of expanding waves of neural activity throughout the network. Conditions for such stable propagation of spiking activity in biological neural circuits have been examined both theoretically (Diesmann et al., 1999; Kumar et al., 2008, 2010) and experimentally (Reyes, 2003; Wu et al., 2008; Nauhaus et al., 2012). Recent electrophysiological results suggest existence of expanding waves of neural activity in the hippocampus during, so called, sharp wave ripple (SWR) episodes (Ellender et al., 2010). Sharp wave ripples are brief high-frequency bursts of neural activity observed during sleep or at awake rest (Buzsaki, 1986). Hippocampal SWRs are frequently accompanied by sequential reactivation of place cells occuring in the same- or reverse temporal order as previously experienced during behavior, but replayed at a compressed time scale (Pavlides and Winson, 1989; Wilson and McNaughton, 1994; Foster and Wilson, 2006). Interestingly, reactivation patterns observed in the awake animals are not always just a simple function of experience (Gupta et al., 2010), and have also been reported to represent trajectories never directly or fully experienced by an animal, suggesting a possible role of the awake SWRs in planning, navigation or decision making (Pastalkova et al., 2008; Buhry et al., 2011; Foster and Knierim, 2012; Singer et al., 2013). These results point to the awake-state SWRs as a possible biological candidate process for parallel mental exploration as required in our model. Moreover, it has been suggested that the SWRs provide optimal conditions for the activation of synaptic plasticity processes, such as STDP (Sadowski et al., 2011)—which, again, is consistent with our assumption that a propagating wave of neural activity should be able to modify connectivity within the network in order to create structured SVFs.

The SVFs are in turn used in our model to guide behavior. Indeed we assume that the movement of an agent (an animal) is guided by the activity of places cells surrounding the present agent location. Therefore, the problem is to generate motor forces which will bring into better alignment two “bumps” of neural activity, one coming from the sensory system representing the actual location of the agent, and the other clump of neural activity having a location biased by the modified synapses. In our paper, this problem is solved by a mathematical algorithm (cf. Methods). However, neurophysiological experiments suggest that the same problem can also be solved by a biological neural network, for it is isomorphic to the problem of moving the two eyes so that the image of one bright spot is centered on both fovea (Ohzawa et al., 1990, 1997). A relatively inefficient but fully neural solution to this two-bump problem was given in (Hopfield, 2010).

As mentioned already, generation of directed connections for SVFs requires asymmetric STDP rules. Such asymmetry in the STDP learning windows has been found in the synaptic connections between hippocampal cells, first in cultured cells (Bi and Poo, 1998) and more recently also in slice preparations (Aihara et al., 2007; Campanac and Debanne, 2008).

“Anti-” or “reverse-” STDP, in which a pairing of a pre-synaptic spike that precedes a post-synaptic spike *decreases* the strength of a synapse (Bell et al., 1997; Kampa et al., 2007), was used in our model to produce the SVF. There are two important reasons for why “normal” (or “pro”) STDP cannot be used in the model. If parameters are set in the fashion of (Hopfield, 2010) so that a clump of activity, once initiated by sensory input, is stable when sensory input is removed, that clump of activity will move, following the vector field. Thus, when the “anti” sign is used, the agent can rehearse mentally the chosen trajectory from its present location to the chosen goal. It could even, with slight elaboration, communicate a sequential list of way points. Such a natural behavior of mental rehearsal in sequential order from the starting point is not available with “pro” STDP, for the clump of activity in this case moves away from the target. Initiating a clump of activity at the target location does not create an equivalent in reverse order because the vector field diverges from that point. Another advantage of using anti-STDP over STDP is apparent for navigation in the presence of neural noise or external perturbation (physical forces pushing the agent away from the original path). When using anti-STDP, flow field lines converge when looking toward the source of the expanding circular wavefront that generated the field. When following in this direction, nearby vector field lines all converge toward the same destination, so noise is attenuated by the following process and has little effect. When following away from a source, as would be the case for normal STDP, vector field lines diverge, the effect of a noise error is amplified, and effects of noise accumulate.

Our model assumes that whenever a new planning process is necessary, all synapses are reset to the baseline state and waves of activity can be initiated from the present target locations to create new SVFs. There are several candidate phenomena observed in the nervous system that could potentially realize the necessary resetting mechanism. One hypothesis, that seems to have both theoretical and experimental support, is that the population bursts during sharp wave ripples could serve this task by desynchronizing neurons through STDP (Mehta, 2007; Lubenov and Siapas, 2008). If this is the case indeed, the SWR episodes in our model would need to serve both tasks: memory erasing (hypothetically during the synchronious activation of populations of neurons) and formation of new memories (during the reactivation). To the best of our knowledge though, no such double-function of the SWR has been reported in the experimental literature so far. Another hypothetic mechanism for resetting synaptic connectivity in the hippocampus is through the neuromodulators. For example Bouret and Sara (2005) point to the role of noradrenaline in reorganizing the network structure in a way necessary for memory erasing.

We recognize that not all mechanisms proposed in our work have experimental support from the studies on hippocampus. Hence, biological relevance of our model remains hypothetical. Nevertheless, we believe our approach is useful as a conceptual model, laying grounds for efficient parallel neural computation for navigation and path planning.

### Outlook

Our model can be usefully expanded in many ways. As mentioned before, different costs can be associated with the particular pathways or spatial locations through the uneven distribution of place cells and/or uneven distribution of strength of synaptic connections. This will affect the speed and the shape of the particular wavefronts, and consequently will determine the boundaries of the basins of attraction and best path within each basin.

Giving an animal the ability to actively control the speed of the wavefront propagation through the different regions of the network would provide a way to encode certain features of the environment in the path planning algorithm. Imagine that there is a cost associated with a certain path, e.g., an animal has to go through a “hazardous” area. This cost can be represented in the network through relatively weaker or “shorter” connections between neurons along this path. As a consequence, a wavefront will have a lower velocity when propagating through the place cells associated with this path, making the choice of this pathway less likely. Another possible way to dynamically control the local speed of the wavefront propagation as a function of environmental features, is by enabling interactions of the mental map considered in our present model with other mental maps, each one encoding for different features of the same environment. In this case, mental selection of particular path planning criteria (for example, “find the shortest/the fastest/the safest path”) would activate interactions between the “path planning map” and the appropriate feature maps. These interactions could be implemented through the local excitatory or inhibitory feedback loops between the “path planning” map and the selected “feature maps,” triggered by the propagating wavefront and resulting in the local changes of neuronal excitability, and so of the wavefront propagation speed in the “path planning map.”

In our model we use place cells distributed uniformly, having a single spatial scale, and a simple place field in each of several separate environments. None of these are literally true in the hippocampus. However, by being an oversimplified idealization, it has allowed an exploration of rapid computational possibilities in a network that perhaps over-represents space, and seems a profligate use of neurons. An interesting extension of our work could be a hierarchical model, where space (or more generally memories) would be represented by different groups of neurons at different levels of abstraction.

Several recent studies suggest that the hippocampus can encode memories at multiple levels of “resolution,” from a detailed rendition of specific places or events within a single experience, to a broad generalization across multiple environments or experiences (Steinmetz et al., 2011; Komorowski et al., 2013). Indeed, when we think about our own experience, we seem to be using a context-dependent switching between different representations of space. For example, when we plan to drive from our present location to another place in a town, we typically only focus on specific points in space when decisions about further route need to be taken (e.g., “turn left or turn right”)—at this point we typically don't think about the details of a highway we drive on, but rather on “when and where to turn or what exit to take.” To the contrary, when we need to change a lane on a highway, we quickly switch to the “high-resolution” local map and we use a spatial map of our surround to navigate between other cars and objects. A similar mechanism could be used in an extension of our model to increase efficiency of the implementation and to reduce the demand on resources (number of neurons), without compromising performance and robustness of computation.

From the application point of view our neural model can be extended to the path planning problems in systems with more than two dimensions or in tasks with extra constraints, such as, e.g., non-holonomic navigation, arm movement planning. Our model, as a particular implementation of the wavefront expansion algorithm, can also be used for solving variety of optimality problems from other domains than motor control (Dorst et al., 1991; LaValle, 2006).

## Methods

The place cell models considered in the paper have been simulated using adapting leaky integrate and fire neurons. The dynamics of the neuron models between spikes are defined by the following formula:

(1) $$\begin{array}{l} \tau_{m}\frac{du_{m}(t)}{dt}=-\left(u_{m}(t)-u_{r}\right)\\ \;+R_{m}(i_{\text{sens}}(t)+i_{\text{syn}}(t)+i_{\text{ns}}(t)-i_{\text{inh}}(t)-i_{\text{Ca}}(t)), \end{array}$$

(2) $$\tau_{\text{Ca}}\frac{di_{\text{Ca}}(t)}{dt}=-i_{\text{Ca}}(t),$$

where *u*_*m*_(*t*) is the membrane potential, τ_*m*_ = *C*_*m*_*R*_*m*_ is the membrane time constant, *C*_*m*_ = 1 nF and *R*_*m*_ = 20 MΩ are the membrane conductance and resistance, respectively, *u*_*r*_ = 0 mV is the membrane potential at rest, *i*_sens_(*t*) is the sensory input, *i*_syn_(*t*) is a sum of the currents supplied by the particular excitatory synapses entering the given neuron, *i*_ns_(*t*) is the non-specific background current modeled as a gaussian process with zero mean and variance 5 nA, *i*_inh_(*t*) is the global inhibitory current, *i*_Ca_(*t*) represents a neuron-specific inhibitory current that could be caused by calcium-activated potassium channels in real neurons.

The neuron produces an instantaneous action potential when *u*_*m*_(*t*) reaches a threshold of 10 mV, and then *u*_*m*_(*t*) is reset to 0 and held at that value for 2 ms to produce an absolute refractory period. Each action potential produced by the neuron allows for a momentary burst of calcium (Ca^2+^) ions to flow into the cell (through high-potential Ca2+ channels) and increments *i*_Ca_(*t*) upward. Calcium ions also leak out, with a characteristic time τ_Ca_ usually set at 1–5 s. Because *i*_Ca_(*t*) and the internal Ca^2+^ ion concentration of the neuron are proportional, the adaptive effect can be written in terms of the variables *i*_Ca_(*t*), and the cellular internal Ca^2+^ concentration is needed only to understand a possible mechanism of spike-frequency adaptation. The timescale of adaptation is set by the size of increment to *i*_Ca_(*t*) that occurs when a neuron spikes.

**Figure 8:**
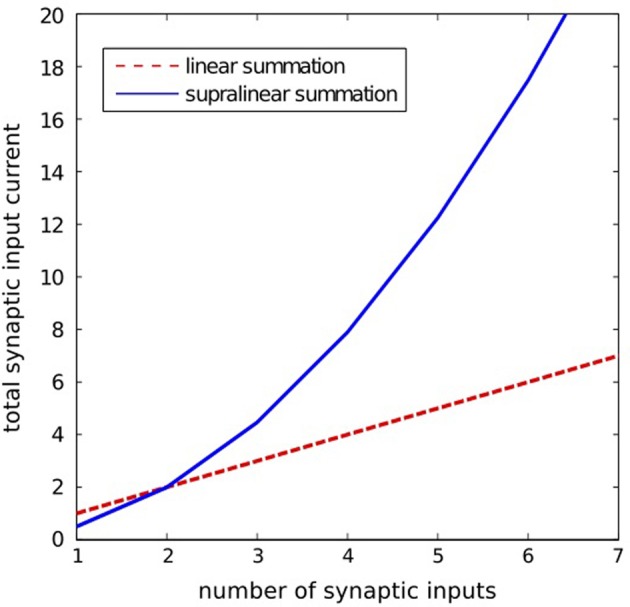
**lllustration of the supralinear and linear summation.** The supralinear function is given by Equation 3. The linear summation function is defined by: *i*_syn_(*t*) = Σ_*j*_ w_*j*_i_*j*_(*t*). Here, for the supralinear function we took *a*_syn_ = 10, *b*_syn_ = 0.05, and for both functions we assumed *w*_*j*_i_*j*_(*t*) = 1 for all *j*.

For the calculation of the total synaptic currents *i*_syn_(*t*) injected into the particular neurons we use a supra-linear spatial summation model (Nettleton and Spain, 2000; Urakubo et al., 2004). The model favors a near simultaneous activation of a neuron from multiple presynaptic neurons over the activation from a single neuron. This approach is supposed to decrease the probability of initiating random wavefronts arising from isolated spikes in the noisy network. The model for supralinear summation used in our simulations is described by the following equation:

(3) $$i_{\text{syn}}(t)=a_{\text{syn}}=\tanh\text{​}\left(b_{\text{syn}}\sum_{j}H\left(i_{j}(t)\right)\right)\sum_{j}w_{j}(t)i_{j}(t),$$

where *i*_*j*_(*t*) is the synaptic current of the *j*-th input; *w*_*j*_(*t*) is the synaptic strength of the *j*-th input; *H(x)* is the step function [*H*(*x*) = 1 for *x* > 0 and *H*(*x*) = 0 for *x* = 0]; *a*_syn_ and *b*_syn_ are the positive constants. The particular synaptic currents *i*_*j*_(*t*) rise instantaneously and decay exponentially with a 25 ms time constant. The supralinear summation function given by Equation 3 is illustrated in Figure 8.

Sensory currents *i*_sens_(*t*) for each place cell are modeled as having an isotropic Gaussian form around the center of the receptive field for that cell, with the same width and strength for each neuron. When modeling multiple environments, each cell has a receptive field in each environment, assigned randomly.

It is assumed that the modeled network contains a set of inhibitory interneurons whose function is to limit the total activity of the network. Because the inhibitory feedback is assumed to be global, and because this essential function is computationally trivial, its effect is modeled in a continuous fashion and using global variables rather than by using spiking interneurons. Hence the dynamics of inhibitory population are given by the following equations:

(4) $$\tau_{e}\frac{di_{e}(t)}{dt}=-i_{e}(t)+a_{e}\sum_{j}\sum_{f}\delta \left(t-t_{j}^{f}\right).$$

(5) $$\{\begin{array}{ll} A_{\text{inh}}(t)\propto (i_{e}(t)-I_{e0}) & \text{if }i_{e}(t)>I_{e0,}\\ A_{\text{inh}}(t)=0, & \text{otherwise}. \end{array}$$

The variable *i*_*e*_(*t*) represents the input current to the inhibitory population from all excitatory cells in the network, whereas *A*_inh_(*t*) reflects the activity of the inhibitory population. According to (4) the current *i*_*e*_(*t*) decays with a time constant τ_*e*_ and is incremented by *a*_*e*_ by each individual spike fired at time *t*^*f*^_*j*_ (with *f*-being the label of the spike) by any excitatory neuron *j* in the network. The parameters τ_*e*_ and *a*_*e*_ are positive and constant; a Dirac function δ(.) is defined as: δ(*t*) = 0 for *t* ≠ 0 and ∫ δ(*t*)*dt* = 1. According to (5) the population activity *A*_inh_(*t*) is proportional to the current *i*_*e*_(*t*) with a firing threshold *I*_*e*0_. Given the activity *A*_inh_(*t*), the global inhibitory feedback *i*_inh_(*t*) to every excitatory neuron in the network is assumed:

(6) $$i_{\text{inh}}(t)=a_{\text{inh}}A_{\text{inh}}(t),$$

where *a*_inh_ is a binary gating variable. The gating variable *a*_inh_ is set to 1, and accordingly the inhibition is active, during the network exploration or during the navigation task; whereas *a*_inh_ = 0 and the inhibition is deactivated during the wavefront propagation.

A fully connected network with excitatory connections has been assumed in all simulations, with all network connections being initially silent. A typical size of the simulated networks varied from 2000 to 4000 place cells in the particular experiments. The simulations were carried out using an Euler integration of the differential equations and a 0.2-ms time step.

### Synaptic plasticity

Synaptic connections have been altered according to the STDP model described by the following equation [cf. Kempter et al. (1999)]:

(7) $$\frac{dw_{ji}(t)}{dt}=a+d\left[S_{i}(t)\int_{0}^{\infty }a_{ij}(s)S_{j}(t-s)ds+S_{j}(t)\int_{0}^{\infty }a_{ji}(s)S_{i}(t-s)ds\right]\text{​​},$$

where *w*_*ji*_(*t*) is the synaptic coupling from neuron *i* to neuron *j, a* < 0 is the activity-independent weight decay, *S*_*i*_(*t*) and *S*_*j*_(*t*) are the pre- and postsynaptic spike trains, respectively. A spike train is defined as: *S*(*t*) = Σ_*f*_δ(*t*^*f*^ − *t*), where *t*^*f*^ is the *f*-th firing time. The terms *a*_*ij*_(*s*) and *a*_*ji*_(*s*) are the integral kernels, with *s* being the delay between the pre-and post-synaptic firing times (*s* = *t*^*f*^_*i*_ − *t*^*f*^_*j*_). The kernels *a*_*ij*_(*s*) and *a*_*ji*_(*s*) determine the shape of the STDP learning window. In our model we use exponential functions given by (8) to describe the STDP curve, however, other shapes are also possible.

(8) $$\{\begin{array}{ll} a_{ji}(-s)=+A_{ji}\cdot \exp\left(s/\tau_{ji}\right) & \text{if }s\leq 0,\\ a_{ij}(s)=-Aij\cdot \exp\left(-s/\tau_{ij}\right) & \text{if }s>0, \end{array}$$

Here, *A*_*ji*_, *A*_*ij*_ are the amplitudes and τ_*ji*_, τ_*ij*_ are the time constants of the learning window. In our model we assume that *A*_*ji*_ > *A*_*ij*_ > 0 and τ_*ji*_ = τ_*ij*_ > 0. The parameter *d* in (7) controls the polarity of the STDP process and can be linked to the concentration of specific neuromodulators known to be able to change the polarity of the synaptic plasticity in biological synapses (Seol et al., 2007). For simplicity, in our model *d* = {−1,0,1}. We assume that during the environment exploration phase *d* = 1, and consequently the synaptic connections undergo STDP with a positive net effect (because *A*_*ji*_ > *A*_*ij*_). During the wavefront propagaton phase: *d* = − 1 and accordingly the synaptic connections are altered by the reversed STDP rule. No synaptic plasticity is assumed during the movement execution phase (*d* = 0).

### Synaptic vector field illustration

In Figures 4, 5, 8 we present sample SVFs created by the propagating activity wavefronts. These vector fields are illustrated using directed arrows originating from the preferred locations of each place cell in the network. The direction and the length of each arrow represent, respectively, the direction and the strength of the vector field in a given location. Here we describe an algorithm used to illustrate the vector field.

For each neuron *n*_*i*_ in the network consider a set *N*_*ji*_ of all neurons *n*_*j*_ on which *n*_*i*_ makes direct synaptic projections. Now for the neuron *n*_*i*_ we define a vector *r*_*i*_(*t*):

(9) $$r_{i}(t)=\sum_{j}w_{ji}(t)\left(x_{j}-x_{i}\right)\text{​}/\text{​}\sum_{j}w_{ji}(t),$$

We assume that the vector *r*_*i*_(*t*) begins in the preferred location *x*_*i*_ of place cell *n*_*i*_ and ends in a center of gravity of the preferred locations *x*_*j*_ of the neighboring place cells *n*_*j*_ ∈ *N*_*ji*_, weighted by the corresponding connection strengths *w*_*ji*_(*t*).

### Exploration algorithm

An exploration procedure was used to establish a set of synaptic connections appropriate to the topology of a particular environment, based on earlier work (Hopfield, 2010). The trajectory followed was a noisy straight line with constant speed, with a directional persistence length of the same scale as the largest dimension of an environment. The trajectory made a specular bounce when it encountered a wall. During this exploration the place cells had sensory inputs according to their spatial receptive fields. Place field centers were assigned on a regular grid, with Gaussian noise around those locations. Pre-post synaptic spike pairs were accumulated for each intra-place cell synapse during the exploration. The potential for synapse change was evaluated over these spike pairs with a weighting function *dw*_*ji*_(*t*)/*dt* = exp(−|*t*_*i*_ − *t*_*j*_|/τ_*e*_) and used to select which synapses should be established. In the equation, *w*_*ji*_(*t*) is the strength of the synaptic equation from a presynaptic neuron *i* to a postsynaptic neuron *j*; *t*_*i*_ and *t*_*j*_ are the firing times of the pre- and postsynaptic neuron, respectively; τ_*e*_ is the learning time constant. When the exploration is finished, each place cell *j* was given incoming synapses of the same size to the set of *m* neurons with the largest values of weights *w*_*ji*_.

This procedure is insensitive to the details. Since any trajectory could be traversed in either direction, it will yield virtually the same set of synapses over a large range of parameters and variations in the form of *S*, as long as there is a net positive area under the curve *S*, and the exploration is extensive. The resulting connection matrix is similar to that which would be achieved by connecting each place cells to its *m* nearest neighbors.

### Navigation algorithm

Once a vector field is created, a simple motor control algorithm is applied for the animal navigation. The algorithm is performed in the following steps:

1. A receptive field corresponding to the present animal location is activated by applying tonic excitation to the corresponding place cells
2. A weak global, activity-dependent inhibition (cf. Equations 4–6) is applied to suppress random spikes resulting from the background noise or from crosstalk between different environment representations.
3. Every spike observed in the network is supposed to act as an instantaneous attractor causing a pulse of force moving the animal toward the preferred location of the active place cell:
  (10) $$F(t)=a_{F}\sum_{j}\sum_{f}\delta \left(t_{j}^{f}-t\right)\left(x_{j}(t)-x_{a}(t)\right)$$
  (11) $$H(x_{a}){\ddot{x}}_{a}+c(x_{a},{\dot{x}}_{a},F_{\text{ext}})-F=0.$$

Equation 10 defines the force vector *F*(*t*) caused by spikes generated by place cells active at time *t*. Equation 11 describes the dynamics of the animals movement in the physical world. Here *x*_*a*_(*t*), *x*′_*a*_(*t*) and *x*″_*a*_(*t*) are, respectively, the location, velocity and acceleration of the animal's center of mass (for clarity we omitted the symbol *t* in Equation 11); *x*_*j*_—is the preferred location of the place cell *n*_*j*_; as before, *t*^*f*^_*j*_ is the firing time of the *f*-th spike in neuron *n*_*j*_; δ(.) is the Dirac function; *a*_*F*_ is the constant gain, *F*_ext_ denotes all possible external forces acting on the animal, *H* is the inertia matrix and *c* is a bias force (Craig, 2004).

### Conflict of interest statement

The authors declare that the research was conducted in the absence of any commercial or financial relationships that could be construed as a potential conflict of interest.

## Footnotes

^1^We use the term “clump of activity” to refer to a localized neural activity of a cluster of place cells representing nearby spatial locations.

## Supplementary material

Movie S1 **Path planning and navigation in a network of place cells using a wavefront expansion concept**. This movie is related to Figure 3 and illustrates two processes: (1) path planning and (2) navigation. In the first phase—path planning—a wavefront is initiated at the place cells representing the navigational target B. The wavefront propagating through the network modifies synaptic connections and creates a SVF with a single attractor at the location B. Red dots are the action potentials. A simulated animal is initially located at point A. Activation of the place cells at A through the passing wavefront triggers the second phase of the process—the navigation. In this phase, the place cells with receptive fields covering the current animal location receive strong excitatory currents from sensory inputs. These cells are indicated in the movie by green dots. The current animal location is denoted by the yellow circle. The stimulated cells fire and in turn excite neighboring cells. Due to the SVF the active cells excite most strongly these neighbors that are located along an optimal pathway toward the target. A simple motor control algorithm (Equations 10, 11) is used to move the animal toward the locations represented by the firing cells, up to the target location.

Movie S2 **Path planning and navigation in a system with multiple targets**. This movie is related to Figure 4. Three wavefronts are initiated simultaneously at the place cells representing target locations B1, B2, B3. The expanding waves create SVFs with centers corresponding to the points of the wavefront initiation. The wavefronts inhibit each other effectively. The points where the wavefronts meet define borders of the basins of attractions of the particular SVFs. Red dots flashing on the screen represent action potentials. A simulated animal is initially located at point A. Activation of the place cells at A through the passing wavefront triggers the second phase of the process—the navigation. In this phase, the place cells with receptive fields covering the current animal location receive strong excitatory currents from sensory inputs. These cells are indicated in the movie by green dots. The current animal location is denoted by the yellow circle. The stimulated cells fire and in turn excite neighboring cells. Due to the SVF the active cells excite most strongly these neighbors that are located along an optimal pathway toward the nearest target. A simple motor control algorithm (Equations 10, 11) is used to move the animal toward the locations represented by the firing cells, up to the target location.
